# Pigmented Lesion of Buccal Mucosa

**DOI:** 10.1155/2014/936142

**Published:** 2014-08-06

**Authors:** Manas Bajpai, Malay Kumar, Manish Kumar, Deshant Agarwal

**Affiliations:** ^1^Department of Oral and Maxillofacial Pathology, NIMS Dental College, Jaipur, Rajasthan, India; ^2^Department of Oral and Maxillofacial Pathology, Ahmedabad Dental College, Ahmadabad, Gujarat, India; ^3^Department of Prosthodontics, NIMS Dental College, Jaipur, Rajasthan, India

## Abstract

Pigmented lesions are commonly found in the mouth. Such lesions represent a variety of clinical entities, ranging from physiologic changes to manifestation of systemic illness and malignant neoplasm. Diagnosis of such lesions requires a proper case history, extraoral and intraoral examination, and, in some cases, biopsy, aspiration cytology, and laboratory investigations. Here we present a case of purple lesion on the buccal mucosa of a 34-year-old male patient which was provisionally diagnosed as mucocele but on the basis of histopathological picture it was finally diagnosed as angiofibroma, and we also discuss the clinical and histopathological differential diagnosis.

## 1. Introduction

Pigmented lesions are commonly found in the mouth. Such lesions represent a variety of entities, ranging from racial pigmentation to manifestation of systemic illness (Addison's disease) and benign (hemangioma, angiofibroma) and malignant neoplasms (Kaposi's sarcoma) [1].

Angiofibromas are uncommon, highly vascular benign but locally aggressive tumors that characteristically arise within the nasopharynx and are predominantly seen to occur in young adolescent males [2]. Nucci et al. described angiofibroma as an uncommon benign mesenchymal tumor in 1997 [3]. The term extranasopharyngeal angiofibroma has been applied to vascular, fibrous nodules occurring outside the nasopharynx. As on 2009, 56 extranasopharyngeal fibromas have been reported, with the most common site of presentation being the maxillary sinus [4]. Juvenile angiofibroma is the most common benign tumor of nasopharynx. It is believed that juvenile angiofibroma is the testosterone dependent tumor [5]. Pathogenesis of angiofibroma is not very clearly understood. Various predisposing factors have been proposed in literature such as infection, trauma, arteriovenous malformation and hormones [6].

## 2. Case Report 

**Figure 1:**
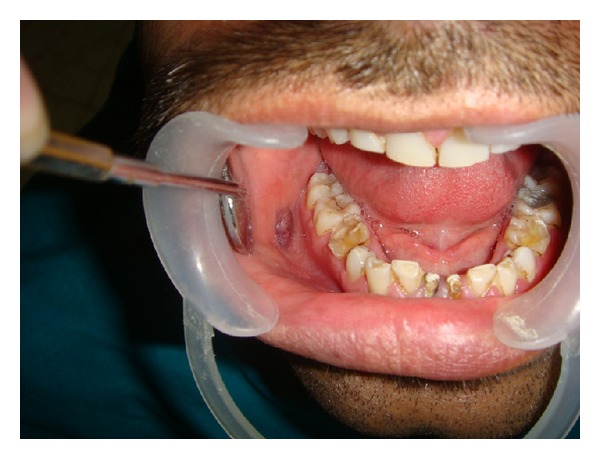
Purple lesion of buccal mucosa.

**Figure 2:**
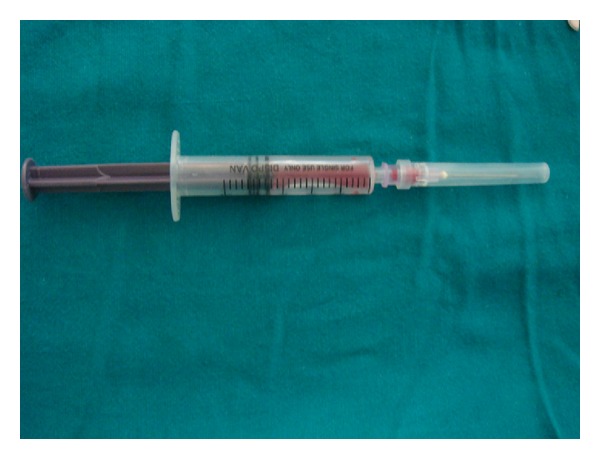
Aspiration consists of blood.

A 37-year-old male patient reported to the Department of Oral Medicine and Radiology with the chief complain of swelling on the right buccal mucosa from last one year. On clinical examination a well circumscribed red to purple lesion in color approximating 2 × 2 cm on the right buccal mucosa was noted (Figure 1). The swelling was firm in consistency and nontender on palpation. The mucosa overlying the swelling was normal in color with absence of any ulceration. There was no history of chronic cheek biting and irritation was reported. Association of any extra oral swelling was not evident. On the basis of clinical features provisional diagnosis of mucocele was made. Aspiration was performed by 25-gauge needle under topical anesthesia (Figure 2). A collection of frank blood was obtained which was subjected to cytological examination. Cytological examination ruled out the possibility of mucocele. Ultrasound reports confirmed that the lesion was not associated with any feeder vessel which is the characteristic feature of hemangioma.

**Figure 3:**
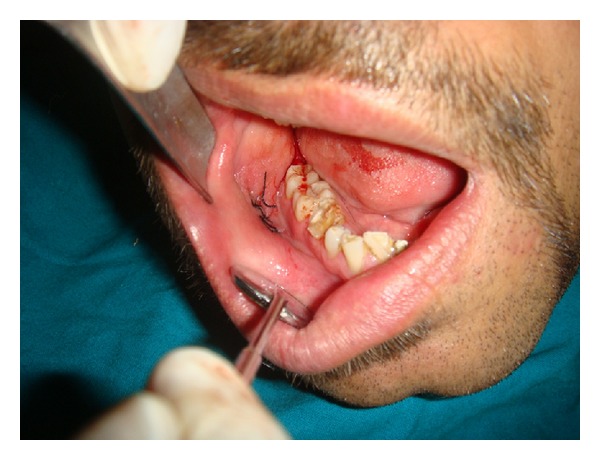
Postoperative picture.

**Figure 4:**
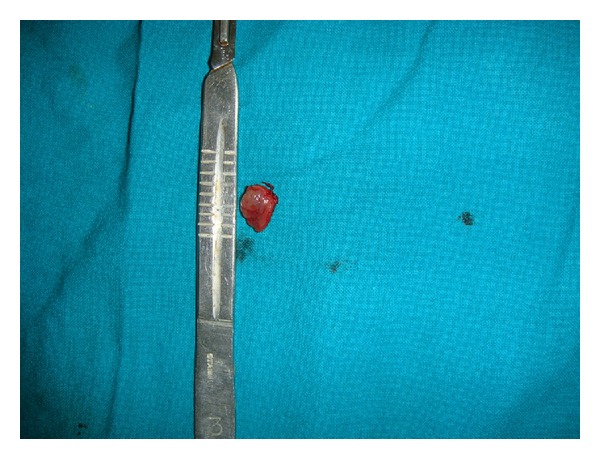
Excised tissue.

After the correlation of clinical, cytological, and ultrasound finding, treatment was planned for surgical excision under local anesthesia (Figure 3). Dissection of the lesion revealed a soft and well-encapsulated swelling. The excised specimen was sent to the Department of Oral and Maxillofacial Pathology for histopathological examination (Figure 4).

**Figure 5:**
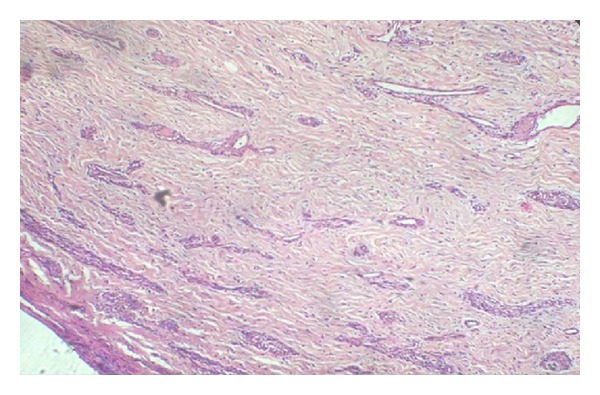
Fibrocellular connective tissue shows proliferating fibroblast and numerous blood vessels (hematoxylin and eosin stain 10x).

**Figure 6:**
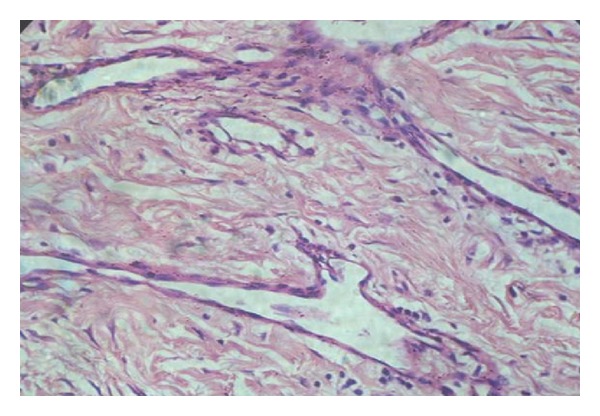
Large sinusoidal spaces surrounded by endothelial cells (hematoxylin and eosin stain 40x).

Histopathological examination revealed fibrous connective tissue composed of numerous collagen fibers and proliferating fibroblasts (Figure 5). Numerous dilated blood vessels and sinusoidal spaces surrounded by endothelial cells were also noted (Figure 6).

On the basis of histopathological features final diagnosis of angiofibroma was made. The follow-up period was uneventful without any recurrence and other complications.

## 3. Discussion

Pigmented lesions are commonly found in the mouth. Such lesions represent a variety of clinical entities, ranging from physiologic changes to manifestation of systemic illness and malignant neoplasm [7]. Hemangioma and mucocele are commonly encountered in oral cavity as pigmented swelling with the color ranging from red to purple. However clinical tests such as diascopy and laboratory investigations such as blood test are useful to reach the definitive diagnosis of hemangioma; on the other hand mucocele is usually associated with a traumatic injury and commonly occurs on the lower lip [8]. Angiofibromas are rare benign mesenchymal tumor characterized clinically by red to purplish swelling and microscopically by bland spindle shaped cells arranged without any pattern in a stroma with wispy collagen and numerous small and medium-sized thick-walled vessels [9].

The present case also exhibited a purple swelling clinically resembling hemangioma or mucocele as the ultrasound reports did not show any feeder vessel so the hemangioma was excluded and aspiration cytology did not yield the mucin so mucocele could also be excluded from the final diagnosis. The microscopic differential diagnosis for CAF includes angiomyolipoma, angiomyofibroblastoma, juvenile nasopharyngeal angiofibroma, solitary fibrous tumor, and spindle cell lipoma [10] (Table 1).

Besides the different location, typical clinical characteristics of extranasopharyngeal angiofibromas, such as, symptoms, age, and sex, do not conform to a great extent with that of nasopharyngeal angiofibroma. This fact has led to doubt as to whether extranasopharyngeal angiofibromas, though structurally similar, should be considered as being different from nasopharyngeal angiofibroma [5]. Angiofibromas are histologically composed of a proliferating vascular component set in a fibrous stroma. The former is characterized by blood vessels of different size and smooth muscle content. Immunohistochemical analysis has shown that stromal cells have strong cytoplasmic reactivity for vimentin and are generally immunonegative for smooth muscle actin [8].

It can be concluded that a definitive diagnosis of angiofibroma is not very difficult but one must need to rule out the possibilities of hemangioma and mucocele on the clinical basis and angiomyolipoma, angiomyofibroblastoma, juvenile nasopharyngeal angiofibroma, solitary fibrous tumor, and spindle cell lipoma on the histopathological basis.

## Figures and Tables

**Table 1:** Differential diagnosis of angiofibroma and specific features [11].

Differential diagnosis	Features
Angiomyolipoma	Collection of fat cells with muscular arteriole
Angiomyofibroblastoma	Loose, myxoid, and fibroblastic element
Juvenile nasopharyngeal angiofibroma	Cornified to the nasopharynx and histopathologically shows homogenous fibroplasias with immature collagen, and sinus-like vascular channels
Solitary fibrous tumors	Do not exhibit prominent vascular elements
Spindle cell lipoma	Adipocytes intimately interspersed with spindle cells
Pyogenic granuloma	Large thin-walled vessels in a loose connective tissue stroma infiltrated throughout by leukocytes
